# Returning Results in the Genomic Era: Initial Experiences of the eMERGE Network

**DOI:** 10.3390/jpm10020030

**Published:** 2020-04-27

**Authors:** Georgia L. Wiesner, Alanna Kulchak Rahm, Paul Appelbaum, Sharon Aufox, Sarah T. Bland, Carrie L. Blout, Kurt D. Christensen, Wendy K. Chung, Ellen Wright Clayton, Robert C. Green, Margaret H. Harr, Nora Henrikson, Christin Hoell, Ingrid A. Holm, Gail P. Jarvik, Iftikhar J. Kullo, Philip E. Lammers, Eric B. Larson, Noralane M. Lindor, Maddalena Marasa, Melanie F. Myers, Josh F. Peterson, Cynthia A. Prows, James D. Ralston, Hila Milo Rasouly, Richard R. Sharp, Maureen E. Smith, Sara L. Van Driest, Janet L. Williams, Marc S. Williams, Julia Wynn, Kathleen A. Leppig

**Affiliations:** 1 Vanderbilt Clinical and Translational Hereditary Cancer Program, Vanderbilt-Ingram Cancer Center, and Department of Medicine, Vanderbilt University Medical Center, Nashville, TN 37232, USA; 2 Genomic Medicine Institute, Geisinger, Danville, PA 17822, USA; akrahm@geisinger.edu (A.K.R.); jlwilliams3@geisinger.edu (J.L.W.); mswilliams1@geisinger.edu (M.S.W.); 3 Department of Psychiatry, College of Physicians and Surgeons, Columbia University, New York, NY 10027, USA; psa21@columbia.edu; 4 Department of Obstetrics and Gynecology, Feinberg School of Medicine, Northwestern University, Chicago, IL 60611, USA; s-aufox@northwestern.edu; 5 Department of Biomedical Informatics, Vanderbilt University Medical Center, Nashville, TN 37232, USA; sarah.bland@vumc.org; 6 Department of Medicine Brigham and Women’s Hospital and Harvard Medical School, Boston, MA 02115, USA; cblout@bwh.harvard.edu; 7 Harvard Pilgrim Health Care Institute and Harvard Medical School, Boston, MA 02115, USA; Kurt_christensen@harvardpilgram.org; 8 Department of Pediatrics and Department of Medicine, Columbia University, New York, NY 10027, USA; wkc15@cumc.columbia.edu; 9 Center for Biomedical Ethics and Society and Department of Pediatrics, Vanderbilt University Medical Center, Nashville, TN 37232, USA; ellen.clayton@Vanderbilt.Edu; 10 Division of Genetics, Department of Medicine, Brigham and Women’s Hospital, Broad Institute of MIT and Harvard, Cambridge, MA 02142, USA; rcgreen@bwh.harvard.edu; 11 Center for Applied Genomics, Children’s Hospital of Philadelphia, Philadelphia, PA 19104, USA; HARRM@email.chop.edu; 12 Kaiser Permanente Washington Health Research Institute, Kaiser Permanente of Washington, Seattle, WA 98101, USA; Nora.B.Henrikson@kp.org (N.H.); Eric.B.Larson@kp.org (E.B.L.); ralston.j@ghc.org (J.D.R.); 13 Center for Genetic Medicine, Northwestern University, Chicago, IL 60611, USA; christin.hoell@northwestern.edu; 14 Division of Genetics and Genomics, Boston Children’s Hospital and Department of Pediatrics, Harvard Medical School, Boston, MA 02115, USA; Ingrid.Holm@childrens.harvard.edu; 15 Departments of Medicine, Division of Medical Genetics and Genome Sciences, University of Washington, Seattle, WA 98195, USA; gjarvik@medicine.washington.edu; 16 Department of Cardiovascular Medicine, Mayo Clinic, Rochester, MN 55902, USA; kullo.iftikhar@mayo.edu; 17 Department of Internal Medicine, Meharry Medical College, Nashville, TN 37208, USA; Philip.Lammers@bmg.md; 18 Department of Health Sciences Research, Mayo Clinic Arizona, Scottsdale, AZ 85259, USA; nlindor@mayo.edu; 19 Department of Medicine, Division of Nephrology, Columbia University, New York, NY 10032, USA; mmarasa@gmail.com (M.M.); hm2673@cumc.columbia.edu (H.M.R.); 20 Department of Pediatrics, Cincinnati Children’s Hospital Medical Center, College of Medicine, University of Cincinnati, Cincinnati, OH 45229, USA; Melanie.Myers@cchmc.org; 21 Department of Medicine and Department of Biomedical Informatics, Vanderbilt University Medical Center, Nashville, TN 37232, USA; josh.peterson@vanderbilt.edu; 22 Department of Pediatrics, Cincinnati Children’s Hospital Medical Center, University of Cincinnati, Cincinnati, OH 45229, USA; cindy.prows@cchmc.org; 23 Biomedical Ethics Program, Mayo Clinic, Rochester, MN 55902, USA; sharp.richard@mayo.edu; 24 Department of Medicine, Feinberg School of Medicine, Northwestern University, Chicago, IL 60611, USA; m-smith6@northwestern.edu; 25 Department of Pediatrics and Department of Medicine, Vanderbilt University Medical Center, Nashville, TN 37232, USA; sara.van.driest@vumc.org; 26 Department of Pediatrics, Columbia University, New York, NY 10027, USA; jw2500@cumc.columbia.edu; 27 Genetic Services and Kaiser Permanente Washington Health Research Institute, Kaiser Permanente of Washington, Seattle, WA 98101, USA; leppig58@icloud.com

**Keywords:** genomic medicine, genetic testing, return of results, electronic health record

## Abstract

A goal of the 3rd phase of the Electronic Medical Records and Genomics (eMERGE3) Network was to examine the return of results (RoR) of actionable variants in more than 100 genes to consenting participants and their healthcare providers. Each of the 10 eMERGE sites developed plans for three essential elements of the RoR process: Disclosure to the participant, notification of the health care provider, and integration of results into the electronic health record (EHR). Procedures and protocols around these three elements were adapted as appropriate to individual site requirements and limitations. Detailed information about the RoR procedures at each site was obtained through structured telephone interviews and follow-up surveys with the clinical investigator leading or participating in the RoR process at each eMERGE3 institution. Because RoR processes at each of the 10 sites allowed for taking into account differences in population, disease focus and institutional requirements, significant heterogeneity of process was identified, including variability in the order in which patients and clinicians were notified and results were placed in the EHR. This heterogeneity in the process flow for eMERGE3 RoR reflects the “real world” of genomic medicine in which RoR procedures must be shaped by the needs of the patients and institutional environments.

## 1. Introduction

Decreasing costs of DNA sequencing, coupled with an increasing understanding of the human genome, are some of the driving forces behind the acceleration of genomic medicine [1,2,3]. Genomic sequencing is more frequently being performed to diagnose rare disorders, to individualize cancer treatments, and inform drug selection and dosing, as well as to screen for disease susceptibility and carrier status for recessive conditions [4,5,6]. As a result, patients with “medically actionable” conditions and predispositions are being identified, either as a primary findings from clinical testing, or as unanticipated secondary findings unrelated to the initial indication that prompted the sequencing tests [7,8]. In some cases, these results are not specifically ordered or requested by the patients’ healthcare providers (HCPs), who may be unclear about their obligations to interpret and respond to these “unsolicited” findings [9,10].

The rapid pace of testing has led to the current healthcare climate, where patients and their HCPs are increasingly confronting the potential healthcare implications of unanticipated incidental or unsolicited genomic sequencing results [9,11]. The American College of Medical Genetics and Genomics (ACMG) has recommended that conditions associated with a minimum set of 59 genes are sufficiently actionable to be returned to the HCP regardless of the testing indication [12,13]. However, there is no standard practice for returning these types of results from genomic studies to patients, research participants, HCPs, or the health care system. Yet, many have argued that individuals should have the opportunity to learn their actionable genetic results along with referral for appropriate clinical follow up [14].

The NIH has sponsored many initiatives to evaluate use of genomics in healthcare, including the Clinical Sequencing Exploratory/Evidence-Generating Research (CSER) Consortium, Clinical Genomic Resource (ClinGen), Implementing Genomics in Practice (IGNITE) network and the electronic Medical Record and Genomics (eMERGE) Network [15,16,17,18]. Among these, the eMERGE Network is addressing the implementation of genomic medicine within the US healthcare system [18]. Established more than 10 years ago, the primary goal of the network has been “to develop, disseminate, and apply approaches to research that combine biorepositories with electronic medical record systems for genomic discovery and genomic medicine implementation research” [19]. Now in the third phase of funding (eMERGE3), the Network consists of 10 clinical sites and 2 sequencing centers across the US. It is focused on sequencing of 109 medically relevant genes, 14 pharmacogenetic (PGx) variants, and additional medically relevant single nucleotide variants (SNVs), in approximately 25,000 study participants [20]. An essential goal of eMERGE3 is the intentional return of results (RoR) of clinically actionable variants to the consenting participants and their HCPs from a curated set of genes and to incorporate those results into the electronic health record (EHR).

There is currently no protocol or practice standards for returning unsolicited genetic tests that are identified as a consequence of clinical care or research programs. eMERGE3 emulates the “real world” of genomic medicine today, in which organizations are independently exploring incorporation of genomics into clinical practice, and provides an ideal setting to study the RoR processes for genomic sequence results that were not solicited by HCPs in a diverse set of healthcare institutions. This paper describes the planned RoR processes independently developed at each of the 10 eMERGE3 sites and examines the similarities and differences in approaches for the disclosure of unsolicited genomic results to participants and their HCPs in order to identify “best practices” for the utilization and return of genomic information within the healthcare system today.

## 2. Materials and Methods

### 2.1. Study Setting

As part of eMERGE3, each site developed plans for (1) disclosing genetic results to patients, (2) disclosing genetic results to HCPs, and (3) incorporating the result in the EHR. The processes and procedures for these elements of RoR were adapted as necessary to individual site context, population, and institutional requirements. Information about the intended RoR processes and protocols was collected from the 10 participating eMERGE3 sites, including Cincinnati’s Children’s Hospital and Medical Center (CCHMC), Children’s Hospital of Philadelphia (CHOP), Columbia University (CU), Geisinger (GE), Kaiser Permanente of Washington (KPWA, formerly Group Health Cooperative)/University of Washington (UW), Mayo Clinic (MC), Meharry Medical College (MMC), Northwestern University (NU), Partners HealthCare (PHC), and Vanderbilt University Medical Center (VUMC) (Table 1). eMERGE3 methods have been described elsewhere [20,21], but briefly, participants were recruited via bio-repositories, health clinics or community settings.

Participant DNA samples were analyzed using the eMERGEseq next generation sequencing platform at either the Partners HealthCare Laboratory for Molecular Medicine (LMM) or the Baylor College of Medicine Human Genome Sequencing Center (HGSC) Clinical Laboratory, both of which are Clinical Laboratory Improvement Amendments (CLIA) and College of American Pathologist (CAP) certified. A minimal set of clinically actionable and returnable genes was determined by expert consensus within the eMERGE Network that consisted of a core set of 68 genes with the addition of genes as designated by each site [20]. The eMERGEseq platform included genes recommended for secondary findings disclosure by the ACMG [12,13], as well as additional genes selected by each site based on research interest such as additional colorectal cancer, cardiovascular, or kidney disease genes [20]. The initial platform was designed to include the “ACMG 56” set, and was later expanded to include 58 of the 59 genes currently recommended by ACMG [13]. Also included were other medically relevant SNVs, including PGx SNVs.

Positive results of the genomic tests (i.e., pathogenic (P) or likely pathogenic (LP); rare variants with presumed major impact on the function of these genes [22]) were slated to be returned to those participants who had consented to receive their eMERGE3 results, their HCPs, and incorporated into the EHR according to each site’s RoR process. The EHR integration has been described [23] where structured results from laboratories were transferred to the site using sFTP and an HTTPS-based Simple Storage Service (S3) file transfer provided by DNAnexus [20]. An open source an XML structured result format was developed which was informed by Health Level 7 (HL7) standards. A PDF file was created for each report that was also uploaded to the participants medical record at the eMERGE site. For the purposes of this manuscript, the RoR process is described after the clinical center receives sequencing report from the molecular laboratory.

### 2.2. Study Analysis

Telephone interviews were conducted by one author (GLW) with the clinical investigator leading or participating in the RoR process at each eMERGE3 institution who had direct and intimate knowledge about the process. Information was obtained for over 30 different factors across the continuum of the RoR process for each eMERGE site. Sites were additionally asked to create a flow diagram of their RoR protocol; several sites had different processes dependent on the age, recruitment or demographic of the population. These flow diagrams were analyzed for sequential order of events, method of RoR communication, such as letter, phone call or clinic appointments, and who disclosed the results to participants. Follow-up information was obtained by surveying the eMERGE RoR Workgroup regarding development of the site’s protocols, and whether there were unanticipated events or alterations that impacted the actual RoR process compared to the initial RoR plan.

Factors identified through the data collection were categorized into one of the three elements relating to the RoR disclosure to the participant at the site, notifying the HCP, or uploading the information to the EHR. Additional descriptors included the demographics of the eMERGE3 participants, the genes and variants that could potentially be released in the genetic test report, the specialty/subspecialty of the provider who would disclose the result to the participant and the timing of the disclosure. Recognizing that both the participant and the HCP need to be informed about the results, the term *disclosure* is used here to indicate how the participant would be informed about the genetic test result, the term *notification* to indicate how the HCP would be informed about the result, and *upload* or *integration* to indicate how the eMERGRE3 report would be placed in the participant’s EHR. HCP is defined in this study as a non-genetic practitioner who has a clinical relationship with the participant, such as primary care provider, non-genetic medical specialist or nurse practitioner.

## 3. Results

### 3.1. Participant Population Characteristics

Similar to the current state of the “real world” of genomic medicine today, in which organizations are independently exploring incorporation of genomics into clinical practice, eMERGE3 sites varied in the recruitment and type of participant enrolled for eMERGE3. To date, each site has enrolled between 500–3000 participants for the eMERGE3 project with a total population of approximately 25,000 participants undergoing sequencing using the eMERGEseq platform. Five of the 10 sites drew samples from both biorepositories and participants recruited specifically for eMERGE3, four sites drew samples from biorepositories only, and one site recruited participants entirely from medical center clinics. Consent for the project was obtained at different time points, including as part of enrollment to the eMERGE study or to an existing biorepository, or when a result was to be released to the participant. The eMERGE3 study population represents all age groups, with two sites enrolling pediatric participants. Table 1 summarizes the expected number of participants, whether the participants were recruited from clinic or samples from an existing biorepository were used, whether they were pediatric or adult participants, and whether the site targeted any specific ancestry/ethnic group or disease phenotype.

### 3.2. Genes Included in RoR

All sites developed plans to return results from a core set of 68 genes on the eMERGEseq platform, however, sites could choose additional genes and types of variants to return since, by design, each site independently developed the RoR processes based on their populations [20]. For example, in addition to the core set of genes, MC returned variants from additional genes related to cardiovascular disease, KPWA/UW returned variants from additional genes related to colon cancer, CU returned variants from additional genes related to kidney disorders, and CU targeted arm added genes causing pediatric autosomal recessive disorders for carrier screening associated with reproductive risks. The sites that enrolled pediatric participants (CCHMC and CHOP) chose to exclude the return of variants in genes associated with adult onset orders, such as breast or colon cancer, from the RoR, except for an adolescent (CCHMC) sub-study participants in which results for some adult onset disorders were offered [24]. One site (PHC) planned to confine RoR to only variants found in the ACMG list of designated genes.

While all 10 sites planned to return P/LP actionable results to their participants [22], sites differed in their plans for the return of variant of uncertain significance (VUS), P/LP heterozygous carriers for autosomal recessive conditions, PGx results, and results without identified P/LP variants, labeled as “negative” or “no variant identified” results (Table 2); thus creating additional variability in the RoR process. Only one site planned to return VUS to a subset of their consenting participants (KPWA/UW). The KPWA/UW site planned to return VUS in mismatch repair genes to participants affected by colon cancer or colon polyps, with subsequent immunohistochemistry (IHC) testing of the tumor tissue if available. Five sites planned to return PGx variants to all or some participants, including a subset of the CCHMC cohort (an adolescent-parent dyad sub-study by participant request), pediatric and adult participants at CHOP, and adult participants at GE, NU and VUMC. Results with no actionable finding (i.e., no P/LP variants in the reportable genes) were planned to be returned to consenting adolescent-parent dyads in the CCHMC sub-study by participant request and to consenting adult participants enrolled at CU, MC, NU, KPWA/UW, and VUMC sites.

One site planned to return results based on sub-study randomization (CCHMC), and two sites allowed participant request (CCHMC, CU). One site (CU-targeted arm) planned to return either P/LP, carrier results or no variant results according to the preferences of participants queried at the time of enrollment. The CCHMC had an adolescent-parent dyad sub-study which planned to return carrier results for selected autosomal recessive conditions by participant request [24]. Further, this group could also choose to learn 14 SNVs primarily related to inborn errors of metabolism as well as some PGx results mentioned previously.

### 3.3. RoR Disclosure Processes

Each eMERGE site independently developed plans for RoR that included three essential components: (1) Disclosure of the results to the participant, (2) notification of the HCP of the result, and (3) integration of the genomic results into the EHR. However, the sites’ plans differed in timing and order of these components, following one of three pathways (Table 3). Five sites intended to disclose the results to the participants first, then notify the HCP followed by upload to the EHR. Three sites intended to disclose the results to the participant first, then upload to the EHR, and notify the HCP. Two sites intended to upload to the EHR first, then notify the provider, and then disclose the results to the participant. Thus, the most common first step in the process was disclosure of the sequencing results to the participant (eight of 10 sites), followed by notification of the HCP and then inclusion in the participant’s EHR (Table 3). Eight sites planned to disclose to the participant as the initial step, and the 2 remaining sites planned to upload to the Electronic Health Record (EHR). Seven sites planned to notify to the healthcare provider (HCP) as the second step.

The eMERGE3 sites developed different RoR processes to communicate results with the participants. Importantly, for the return of positive (P/LP) results, all sites planned to have genetic professionals, either a genetic counselor or geneticist, as either the initial disclosing professional (CCHMC, CHOP, MC, MMC, NU, KPWA/UW), non-genetic specialist or geneticist after the initial disclosure by letter (VUMC), by participant choice at enrollment (CCHMC, CU, GE), or participant choice after learning of an unconfirmed positive result (PHC). One site (CU-targeted arm) planned to conduct sub-study of return that allowed the participant to specify the method of return, such as phone, letter, secure email, or clinic visit. Most sites (CU, CHOP, CCHMC, KPWA/UW, MC, MMC, NU, PHC) planned to contact the participant initially by a letter or a phone call indicating the need for the participant to schedule an appointment with a professional specializing in the results of that gene or their HCP to receive the results. Most sites indicated that they use multiple communication formats, including letter, telephone and/or clinic visits for disclosure and participant communication, with two sites (CCHMC, GE) also planning to use the EHR patient portal. Five sites developed protocols that allowed participants to opt out of receiving all or some of their results (CU-targeted, CCHMC, CHOP, KPWA/UW, PHC).

### 3.4. EHR Integration

Participant reports were integrated into the medical record of the participant by uploading a PDF file. Most sites (8 of 10) elected to upload the report as the last step in the return process. Due to technical issues, structured formatted report data remained in the research data repositories [23]. Several sites identified participants with more than one reportable variant, which required some sites (KPWA/UW, CHOP, VUMC) to manually add additional variant interpretations into the EHR.

## 4. Discussion

The design and structure of the eMERGE3 RoR research network fostered a natural experiment; reflecting the “real world” of genomic medicine today where there are no best practice guidelines and where institutions create processes specific to their organizational structures, populations, and providers. The eMERGE3 network seeks the analysis of up to 100 clinically actionable genes and 14 SNVs of about 25,000 participants across 10 different clinical sites, and requires three elements of return of these results (1) disclosure of the results to the participant, (2) notification of the HCP of the result, and (3) integration of the genomic results into the EHR. Sites were free to choose which genes to return, whether to return VUS and/or “negative” results, and the processes by which return would happen based on their population and institutional requirements and limitations. By analyzing the planned RoR processes, this study describes the complexity and process variation of returning “unsolicited” genomic results to the eMERGE research participants who consented to result disclosure.

Three general pathways were identified across the 10 eMERGE3 sites to deliver the 3 required elements of RoR; with the majority of sites choosing to disclose the genomic results first to the participant and then to notify the HCP. The clinical sites also varied in which additional genes were interrogated and type of variants chosen to disclose to the participants and their HCPs. By design, all eMERGE sites returned P/LP variants in a core set of genes, but differed as to whether to return P/LP variants in additional genes, VUS, carrier state for recessive conditions, or potential mosaicism (Table 2).

Our study results reflect an underlying perception that genetic professionals should be engaged in the RoR process, as 8 of 10 sites planned to have either a genetic counselor or geneticist be the initial provider to disclose the positive results to their participants. This mirrors the experience of other studies, such as CSER study that provided return of secondary findings from genome or exome sequencing from diverse healthcare settings using genetic professionals [15,25]. Concern has been raised, however, about the clinical workforce capacity with the limited number of geneticists and genetic counselors and the much larger number of patients requiring genetic services in the future [26,27,28]. How, and by whom, such results are to be returned is extremely important, particularly in light of the launch of the precision medicine All of Us research project, where up to a million US citizens will undergo genomic testing with disclosure of actionable risk information in the next few years [29].

The traditional model of genetic professionals providing initial disclosure of results will not be scalable in the future, necessitating the development of alternative programs for communicating test results. Studies examining the use of primary care providers in returning results have had promising results [25]. However, it may not be feasible to shift the responsibility for managing genomic test results to primary care providers or other specialists as research has shown that the current HCP workforce lacks the capacity and knowledge to interpret genetic results and manage the disease states linked to actionable gene variants [30,31]. A related eMERGE study has reported that HCPs also have concerns for returning unsolicited results which include lack of knowledge to interpret the test report and negative impact on clinical workflow [9]. Alternative methods for reporting genomic results are being investigated by several groups using web-based informatic technology for patient education [32], and use of telemedicine using video or telephone for counseling [33,34,35].

As genomic testing expands with more clinical and direct to consumer laboratories offering different genomic health screens [7], these diverse initial eMERGE RoR experiences described here can be used as a foundation to better understand the underlying contextual factors, organizational culture, and norms around genomic medicine. Here we have outlined the planned RoR processes as adapted by each of the 10 eMERGE3 sites to address the required elements of (1) disclosing to patients, (2) notifying providers, and (3) integrating the result into the EHR and the additional real-time adjustments to these processes in response to unplanned events early on in the testing process. As the RoR study progresses, the “lessons learned” from the RoR processes at each site will inform current eMERGE3 investigations and other precision medicine projects examining the impact of the RoR on medical outcomes, health care usage, participants and HCPs. For example, a comparative analysis of the three identified RoR processes will be important in understanding the clinical utility, management and institutional resources required for RoR. This will provide further insight into which RoR elements and processes must be considered regardless of organizational context and which processes work best within different institutional structures.

## 5. Conclusions

There is currently no standard of practice for returning unsolicited genetic and genomic data to patients or research participants. This report on initial ROR processes and experiences across 10 eMERGE3 sites highlights the current real-world needs of healthcare systems in developing new pathways to support genomic medicine. By focusing on the required elements of RoR and describing how sites developed processes and procedures to adapt those elements within their unique contexts, we provide the foundation for studying within eMERGE3 and other future studies the impact of these RoR processes on patient, provider, and organization utilization of genomic information.

## Figures and Tables

**Table 1:** Planned enrollment and participant descriptors for 10 sites in the electronic Medical Record and Genomics (eMERGE) network.

Site	Target (n)	Source of Participant ^1^	Age Group	Enriched Population	Enriched Disease Phenotype
CCHMC	2800	Biorepository	Pediatric	None	None
	200 ^2^	Clinic, community	Adolescent	None	None
CHOP	3000	Biorepository	Adult (young) Pediatric	None	Autism, ADHD
CU	500	Community	Adult	Ashkenazi Jewish, Hispanic	None
	1000	Clinic, Community	Adult	None	Renal disease, liver disease and cancer
	1000	Biorepository	Adult	None	Renal disease, liver disease and cancer
GE ^3^	2500	Biorepository	Adult	None	Some tested in research study prior
KPWA/UW	2500	Biorepository	Adult	None	Colorectal polyps and cancer
	500	Clinic	Adult	Asian	None
MC	2500	Biorepository	Adult	None	Hypercholesterol- emia and colon polyps
	500	Biorepository	Adult	Hispanic	Dyslipidemia
MMC	500	Clinic	Adult	African-American	Cancer, or family history of lung, breast, prostate, or colon cancer
NU	3000	Clinic, PGx Biorepository	Adult	None	Lipid disorders, atrial fibrillation, breast cancer, ovarian cancer screening, dermatology
PHC	2500	Biorepository	Adult	None	None
VUMC ^4^	2500	Primary care clinic, PGx Biorepository	Adult	None	None

^1^# Source of participant: Biorepository, Clinic, Community Randomized Trial (RTC). Partial enrollment from previous biorepository research study for ^2^ randomized trial of parent-adolescent dyads, ^3^ whole exome sequencing, ^4^ pharmacogenetic (PGx).

**Table 2:** Type of gene variant planned for disclosure to eMERGE3 participants who consented for RoR by eMERGE site. All sites planned to return pathogenic (P) or likely pathogenic (LP) variants.

Site	Participants	Disclosure	By	Variant Disclosure to Participant				
				P/LP	VUS	Carrier	Pharma	No Variant
CCHMC	Pediatric—Biorepository	Adult onset disorder genes excluded	GC	Yes	No	No	No	No
	Adolescent—Clinic, community	^1^ All, by participant request, RTC	GC or EHR portal	Yes	No	Yes	Yes	Yes
CHOP	Adults—Biorepository	All	GC	Yes	No	No	^2^ Yes	No
	Pediatric—Biorepository	Adult onset disorder genes excluded	GC	^3^ Yes	No	No	^2,3^ Yes	No
CU	Adults—Targeted arm (Clinic, and community)	^4^ All	PAR choice	^4^ Yes	No	Yes	No	^4^ Yes
	Adults—General arm (Clinic, and community)	All	GC	Yes	No	No	No	Yes
	Adults—Biorepository	Re-confirmed	GC	Yes	No	No	No	No
GE	Adults—Biorepository	All	GC	Yes	No	No	Yes	No
KPWA/UW	Adults—Biorepository and clinic	All	GC	Yes	Yes	No	No	Yes
MC	Adults—Biorepository	All	GC	Yes	No	No	No	Yes
	Adults—Clinic	All	GC	Yes	No	No	No	Yes
MMC	Adults—Clinic	All cancer genes and participant’s choice for other genes	GC	Yes	No	No	No	No
NU	Adults—Biorepository	All	GC	Yes	No	No	No	Yes
	Adults—Clinic	All	GC	Yes	No	No	Yes	Yes
PHC	Adults—Biorepository	Re-confirmed	^5^ PAR choice	^4^ Yes	No	No	No	No
VUMC	Adults—PGx and Clinic	All	Letter	Yes	No	No	^2^ Yes	^6^ Yes

Key: Disclosure by a Genetic Counselor (GC), participant (PAR), Healthcare provider (HCP), Letter, or EHR (Electronic Health Record). Sites that enrolled participants: ^1^ Randomized Controlled Trial (RTC) with choices for return; ^2^ Pharmacogenetic variants are uploaded to EHR, and returned by letter; ^3^ Participant choice of type of variant (P/LP or PGx) at time of consent; ^4^ Participant choice after discussion with GC (non-RTC); ^5^ Initial notification of P/LP by GC, but final result disclosed by participant choice; ^6^ Letter sent to participant.

**Table 3:** Order of eMERGE3 site return of results (RoR) for participants consented to return of actionable results.

Number	Sites	First Step	Second Step	Third Step
5	CHOP, PHC ^1^, KPWA/UW ^1^, MMC, NU	Participant	HCP	EHR
3	CCHMC ^1,2^, CU ^1^, MC	Participant	EHR	HCP
2	GE ^3^, VUMC	EHR	HCP	Participant

Key: Healthcare provider (HCP), Electronic health record (EHR). ^1^ Site allowing participants to decline RoR. For CU, the participants in the biorepository arm could decline learning their results. ^2^ Site planned a RTC option to upload to EHR, notify HCP prior to disclosing to participant. ^3^ Concurrent upload to EHR and HCP notification.
